# Power grip, pinch grip, manual muscle testing or thenar atrophy – which should be assessed as a motor outcome after carpal tunnel decompression? A systematic review

**DOI:** 10.1186/1471-2474-8-114

**Published:** 2007-11-20

**Authors:** Jo Geere, Rachel Chester, Swati Kale, Christina Jerosch-Herold

**Affiliations:** 1 School of Allied Health Professions, University of East Anglia, Norwich, UK; 2 Physiotherapy Department, Norfolk and Norwich University Hospital NHS Trust, Norwich, UK

## Abstract

**Background:**

Objective assessment of motor function is frequently used to evaluate outcome after surgical treatment of carpal tunnel syndrome (CTS). However a range of outcome measures are used and there appears to be no consensus on which measure of motor function effectively captures change. The purpose of this systematic review was to identify the methods used to assess motor function in randomized controlled trials of surgical interventions for CTS. A secondary aim was to evaluate which instruments reflect clinical change and are psychometrically robust.

**Methods:**

The bibliographic databases Medline, AMED and CINAHL were searched for randomized controlled trials of surgical interventions for CTS. Data on instruments used, methods of assessment and results of tests of motor function was extracted by two independent reviewers.

**Results:**

Twenty-two studies were retrieved which included performance based assessments of motor function. Nineteen studies assessed power grip dynamometry, fourteen studies used both power and pinch grip dynamometry, eight used manual muscle testing and five assessed the presence or absence of thenar atrophy. Several studies used multiple tests of motor function. Two studies included both power and pinch strength and reported descriptive statistics enabling calculation of effect sizes to compare the relative responsiveness of grip and pinch strength within study samples. The study findings suggest that tip pinch is more responsive than lateral pinch or power grip up to 12 weeks following surgery for CTS.

**Conclusion:**

Although used most frequently and known to be reliable, power and key pinch dynamometry are not the most valid or responsive tools for assessing motor outcome up to 12 weeks following surgery for CTS. Tip pinch dynamometry more specifically targets the thenar musculature and appears to be more responsive. Manual muscle testing, which in theory is most specific to the thenar musculature, may be more sensitive if assessed using a hand held dynamometer – the Rotterdam Intrinsic Handheld Myometer. However further research is needed to evaluate its reliability and responsiveness and establish the most efficient and psychometrically robust method of evaluating motor function following surgery for CTS.

## Background

Carpal tunnel syndrome (CTS) is the most common compression neuropathy, estimated to occur in 4% of the general population [1] with a higher prevalence in women (3% to 5.6%) than men (0.6% to 2.8%) depending on diagnostic criteria used [1,2]. Surgical decompression of the carpal canal is the treatment of choice in moderate to severe cases and accounts for a large number of upper limb surgical procedures. To evaluate the effectiveness of different surgical techniques a range of outcome measures have been used including objective assessment of motor function. Carpal tunnel syndrome presents with a range of symptoms including motor disturbance which can range from weakness of the thenar muscles innervated by the median nerve through to complete paralysis and atrophy. Surgical intervention may reverse these to a greater or lesser extent depending on the severity and duration of the condition [3]. The importance of improved motor function as an outcome may be undisputed however the questions of what parameter should be assessed and with which instrument remain unanswered.

Outcome measures need to be valid for the purpose and population, repeatable over time and across testers and be able to reflect clinically important change [4,5]. There is no lack of available instruments which quantify motor function, however they may not meet the rigorous psychometric criteria required of an outcome measure to the same degree. Whilst several outcome measures are needed to capture the impact of a disorder like CTS on the individual, the use of multiple outcome measures which address the same domain such as motor function should be avoided as it places an unnecessary burden on the patient and clinician.

The primary aim of this review was to identify the methods used to assess motor function following surgical interventions for CTS in published clinical trials and to evaluate the extent to which they reflect clinical change after decompression. A secondary aim was to review those assessments of motor function which reflect change and are psychometrically robust. This may contribute towards a consensus view of how motor function should be assessed in future trials which in turn would facilitate meta-analysis of results [6].

## Methods

A systematic review was conducted to identify randomized controlled trials of surgical interventions for carpal tunnel syndrome which included assessment of motor function as a primary or secondary outcome.

### Search strategy and review criteria

The bibliographic databases Medline [1950 to June 2006], CINAHL [1982 to June 2006] and AMED [1985 to June 2006] were searched using a combination of terms: randomized controlled trial *or *controlled clinical trial, carpal tunnel *and *surgery *or *decompression *or *release. The titles and abstracts of those studies retrieved were read and full-text was obtained for those studies which met the following inclusion criteria: prospective, randomized or quasi-randomized trials, the experimental or comparator intervention included surgical release, the patients had a confirmed diagnosis of carpal tunnel syndrome made through physical examination and clinical history with or without confirmatory electrophysiological testing and the outcomes were described. Only English language publications were considered and irrespective of the year of publication. Studies which assessed motor function through patient-rated questionnaires such as the Boston Carpal Tunnel Questionnaire [7] only were excluded. Each study which met the inclusion criteria was independently read by two reviewers and a data extraction form completed which detailed the type of motor assessment, equipment used, method of assessment, scaling and results.

## Results

A total of 28 studies were identified which met the inclusion criteria. Of these 22 included an assessment of motor function using performance-based tests [8-29]. The other six studies assessed motor function through patient-reported questionnaires only.

The instruments and methods used to evaluate motor outcome following carpal tunnel surgery included i) measurement of grip and pinch strength with dynamometry or vigorimeters, ii) manual muscle strength testing and iii) presence or absence of thenar atrophy.

### Grip and pinch strength measurement

Nineteen of the 22 studies used power grip strength as an outcome measure post surgery. 14 studies assessed both power and pinch grip. Tables 1 and 2 summarise the tools and methods used to assess grip and pinch strength in each study. The amount of detail given on measurement procedure varied greatly and several studies did not state the tool of measurement, the handle position used, number of trials, whether the value reported was a score derived from one attempt, the average or the best of three trials, or the statistical unit of measurement. Only one study reported the positioning of the upper limb during dynamometry [29]. One study [12] adjusted figures for age, sex and gender according to Mathiowetz et al [30].

**Table 1:** Summary of studies evaluating power grip

**Study**	**Instrument**	**Unit of measurement**	**Handle position**	**Patient positioning**
Agee et al., 1992 [9]	Jamar	%	all 5 settings	NR
Bhattacharya et al., 2004 [28]	Jamar	%	2	NR
Brown et al., 1993 [10]	Jamar	lbs	NR	NR
Brüser et al., 1994 [18]	Jamar	%	NR	NR
Citron and Bendall, 1997 [16]	Martin Vigorimeter	kpa	NR	NR
Dias et al., 2004 [29]	Jamar	kg	NR	Y
Dumontier et al., 1995 [14]	Jamar	kg	NR	NR
Erdmann, 1994 [11]	Jamar	lb	NR	NR
Ferdinand and Maclean, 2002 [22]	Baseline hydraulic	Ib	NR	NR
Foulkes et al., 1994 [12]	Jamar	lb	2^2^	NR
Helm and Vaziri, 2003 [26]	Baseline hydraulic	kg	NR	NR
MacDermid et al., 2003 [24]	digit grip device^1^	kg	NR but cites refs	NR but cites refs
Mackenzie et al., 2000 [19]	Baseline hydraulic	kg	2	NR
Mackinnon et al., 1991 [8]	NR	kg	NR	NR
Nakamichi & Tachibana, 1997 [17]	NR	kg	NR	NR
Saw et al., 2003 [25]	Jamar	kg	NR	NR
Sennwald & Benedetti., 1995 [13]	Jamar	kg	2	NR
Trumble et al., 2002 [20]	Jamar	kg	all 5 settings	NR
Wong et al., 2003 [27]	NR	%	NR	NR

^1^NK Biotechnical Corp, Minneapolis, MN, USA;

^2 ^data adjusted for age, sex, and side.

% = percent change from preoperative value

NR = not reported

lb = pounds

kpa = kilopastels

kg = kilograms

**Table 2:** Summary of studies evaluating pinch grip

**Reference**	**Instrument**	**Unit of measurement**	**Protocol**	**Position**	**Key Pinch**	**Tip Pinch**	**Tripod Pinch**
Agee et al., 1992 [9]	NR	%^2^	NR	NR	✓	✓	
Brown et al., 1993 [10]	JAMAR	lb^3^	NR	NR	✓		
Brüser et al., 1994 [18]	B	%	NR	NR	✓	✓	
Dias et al., 2004 [29]	JAMAR	kg	NR	NR		✓	
Erdmann, 1994 [11]	JAMAR	lb	NR	NR	Not specified		
Ferdinand & Maclean, 2002 [22]	B&L	NR	NR	NR	✓		
Foulkes et al., 1994 [12]	B&L	lb	NR	NR	✓	✓	✓
MacDermid et al., 2003 [24]	pinch device NK^1^	kg	NR^3^	NR^3^	✓		✓
Mackenzie et al., 2000 [19]	B&L	kg	NR	NR	✓		
Mackinnon et al., 1991 [8]	NR	NR	NR	NR	Not specified		
Nakamichi & Tachibana 1997 [17]	NR	kg	NR	NR	✓		
Sennwald & Benedetti, 1995 [13]	B&L	lb	NR	NR	✓		
Trumble et al., 2002 [20]	pinch meter^2^	kg	NR	NR	✓		✓
Wong et al., 2003 [27]	NR	%	NR	NR	Not specified		

Total					10	4	3

% = percent change from preoperative value

NR = not reported

lb = pounds

kg = kilograms

B&L pinch gauge (B&L Engineering, Santa Fe, CA)

^1^NK Biotechnical Xorp, Minneapolis, MN

^2^Therapeutic instruments, Clifton, New Jersey

^3 ^Reference to reliability studies provided.

**Figure 1:**
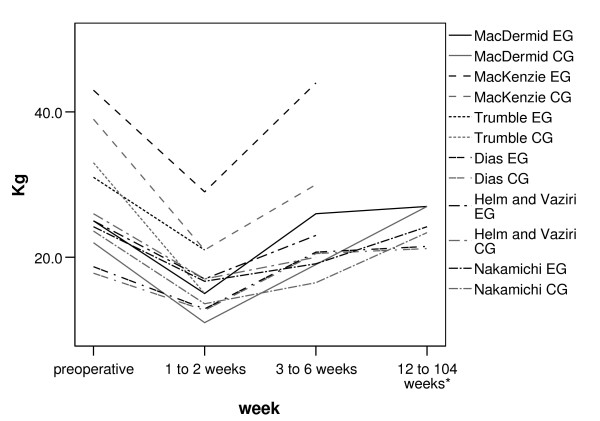
**Line graph of pre and post-operative power grip strength**. EG = experimental group, CG = comparator group. * studies which measured power grip at more than one time point between 12 and 104 weeks demonstrated minimal change in values.

In order to compare the overall magnitude of change across these studies pre-operative and post-operative values from each study were extracted. Of the 19 studies which assessed power grip strength, only six studies [17,19,20,24,26,29] reported actual pre-operative, early and late post-operative values which have been plotted on a line graph (Fig 1).

Figure 1 shows a marked decline in power grip within the first 2 weeks post surgery. By 6 weeks power grip had recovered to, or close to, pre-operative levels. Three further studies [11,13,16] which displayed grip strength graphically only showed a similar trend for values up to 12 weeks post-operatively. Whilst these nine studies together demonstrate similar patterns of recovery, trends in the studies reporting later recovery (greater than 12 weeks) [8,9,11-14,16,17,20,22,24,25,27,29] were less consistent. The differences in later results did not appear to be related to the time at which final post operative assessment took place.

**Figure 2:**
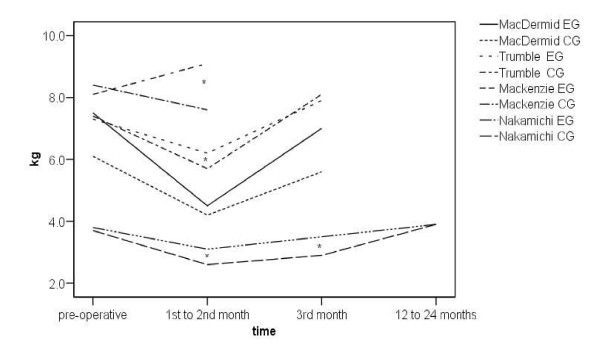
**Line graph of pre and post-operative key pinch strength**. * Statistically significant difference between groups reported. EG = experimental group, CG = comparator group.

**Figure 3:**
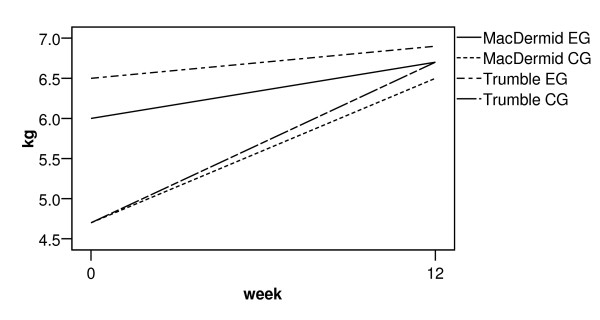
**Line graph of pre and post-operative tripod pinch strength**. Trumble et al. [20] reported a significant difference (p < 0.05) between groups until the 3^rd ^post-operative month but does not report actual 2 week values. MacDermid et al. [24] reported no significant differences between groups. EG = experimental group, CG = comparator group.

**Figure 4:**
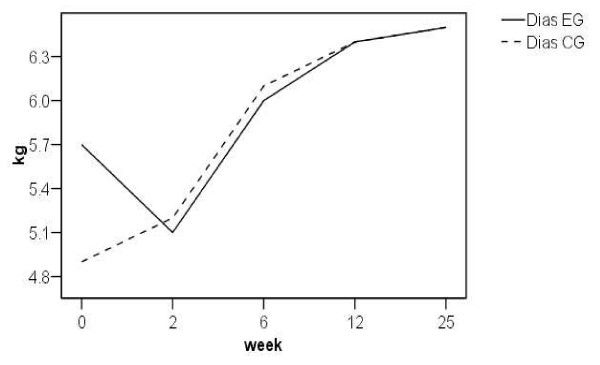
**Line graph of pre and post-operative tip pinch strength**. EG = experimental group, CG = comparator group.

Key, tip and tripod pinch were similarly analysed. Figures 2 to 4 show the change from pre-operative to early post-operative pinch strength for key pinch, tripod and tip pinch, respectively. Only studies where actual values were reported have been included. There was a similar pattern to that observed with power grip, reporting an early post-operative decrease in pinch strength. The trend was for an increase in tip and tripod pinch strength at 12 weeks compared to pre-operative values.

In order to compare the relative responsiveness of power and tip pinch effect sizes were calculated. Effect size (mean change divided by standard deviation of initial score [31]) is a standardised score which is unit free and allows comparison between different scales and also between studies. However several of the studies included in this review did not report means or standard deviations, therefore it was not possible to calculate an effect size from the data given. The severity of motor weakness may also differ within study and between study populations and a high degree of heterogeneity is likely to result in a higher standard deviation of the baseline score (the denominator in the equation) which could result in a small effect size. It was therefore decided to only calculate and compare the effect sizes within studies, that is where both power and tip or tripod pinch were assessed on the same sample and where the data for this could be extracted from the article.

Only two studies [17,29] included both grip and pinch strength and also reported means and standard deviations or 95% confidence intervals for pre- and post-operative assessments. Tables 3 and 4 give the effect sizes for grip and pinch strength at six and 12 weeks post-operatively. In the study by Dias et al [29] the responsiveness of power grip is low indicated by a small effect size of 0.22 or less. Tip pinch strength scores show moderate to large effect sizes suggesting that it is more responsive to change than power grip. The effect sizes in Nakamichi and Tachibana's [17] study are moderate to large in both power and pinch strength however reflect a change in the wrong direction, that is at 6 weeks and 12 weeks both power and key pinch had deteriorated from pre-operative values. Even at the final follow-up which in Nakamichi and Tachibana's [17] study was as long as 2 years the mean scores for power returned to pre-operative values only. Lateral pinch was only slightly improved at 2 years after surgery.

**Table 3:** Responsiveness of power grip (effect sizes)

**Power grip**	Nakamichi & Tachibana 1997 [17]		Dias et al 2004 [29]	
	CG	EG	CG	EG
Mean pre-op/post-op at 6 weeks (kgs)	23.6/16.5	24.2/19.10	17.8/18.5	18.7/17.8
Effect size	1.69*	0.85*	0.06	0.08
Mean pre-op/post-op at 12 weeks	23.6/19.4	24.2/21.6	17.8/20.5	18.7/20.7
Effect size	1.00*	0.43*	0.22	0.18

CG = comparator group, EG = experimental group

* large effect size but change reflects a decrease in strength

**Table 4:** Responsiveness of key and tip pinch grip (effect sizes)

	**Key pinch**		**Tip pinch**	
	Nakamichi & Tachibana 1997 [17]		Dias et al 2004 [29]	
	CG	EG	CG	EG
Mean pre-op/post-op at 6 weeks (kgs)	3.74/2.58	3.84/3.10	4.9/6.1	5.7/6.0
Effect size	1.02*	0.64*	0.46	0.14
Mean pre-op/post-op at 12 weeks	3.74/2.91	3.84/3.48	4.9/6.4	5.7/6.4
Effect size	0.73*	0.31*	0.58	0.33

CG = comparator group, EG = experimental group

* large effect size but change reflects a decrease in strength

### Manual Muscle Testing

Eight of the 22 studies reviewed used manual muscle testing as an outcome measure, five of these included it as well as power or pinch strength [8-10,15,17,20-22]. Table 5 summarises the scale, grading system and muscle(s) tested in each study. Comparison of results was not possible due to the different scales used and several studies did not report pre- and post-operative values. The Abductor Pollicis Brevis (APB) muscle was tested in six studies [8,10,15,17,20,22], two studies [9,21] did not specify the muscle tested. Three studies gave descriptive statistics for pre- and post-operative values [10,15,17]. In Nakamichi and Tachibana's [17] study the 2 year follow-up values had improved from a mean grade of 2.5 (± 2.1) to 3.9 (± 1.9) in the experimental group and from 2.3 (± 2.0) to 4.3 (± 1.7) in the comparator group. A grading of 0–5 was used however no reference is made to which classification system was used. Brown et al [10] used the AOA criteria and a grading of 0 to 5. Mean pre-operative grades were 4.4 and 4.5 for comparator and experimental groups respectively, increasing to 4.6 to 4.7 post-operatively.

**Table 5:** Summary of studies using Manual Muscle Testing (MMT)

			**Muscle tested**		
				
**Study**	**Scale**	**Gra-ding**	**APB**	**OP**	**Results reported as**
Agee et al., 1992 [9]	MRC	1–5	not specified		% pf patients testing normal
Brown et al. 1993 [10]	AOA	0–5	✓		Mean and SD pre-op and post-op
Ferdinand & Maclean 2002 [22]	MRC	0–5	✓	✓	NR
Leinberry et al., 1997 [15]	NR	3–5	✓		Mean and range pre-op and post-op
Mackinnon et al., 1991 [8]	MRC	0–5	✓	✓	Number of hands
Nakamichi & Tachibana 1997 [17]	NR	0–5	✓		Mean and SD pre-op and post-op
Shum et al., 2002 [21]	NR	NR	not specified		NR
Trumble et al., 2002 [20]	AOA	0–5	✓		NR

MRC = Medical Research Council

NR = Not Reported

SD = Standard Deviation

AOA = American Orthopedic Association

APB = Abductor Pollicis Brevis

OP = Opponens Pollicis

### Assessment of thenar atrophy

Presence of thenar atrophy was reported in 5 studies [8,10,20,21,23] (see Table 6). Thenar atrophy can only be assessed subjectively as being present or absent. A four-point categorical scale was used in two studies [8,10]. The overall trend was, as expected, a decrease in proportions of those with thenar atrophy.

**Table 6:** Summary of studies that used thenar atrophy as an outcome measure

**Study**	**Scale**	**Grading**
Borisch et al 2003 [23]	NR	NR
Brown et al 1993 [10]	0–3	0(absent), 1(mild) 2(moderate), 3(severe)
Mackinnon et al 1991 [8]	NR	None, mild, moderate and severe
Shum et al 2002 [21]	NR	NR
Trumble et al 2002 [20]	NR	Present/absent

NR = Not Reported

## Discussion

The primary aim of this review was to identify what methods and instruments have been used to assess motor outcome and to examine their usefulness as an indicator of change over time. Dynamometry of power and pinch grip, manual muscle testing and presence or absence of thenar atrophy, were the three main methods of objectively quantifying motor outcome. Subjective rating of weakness is also included in some of the patient-oriented outcome measures, for example the Symptom Severity Scale of the Boston Carpal Tunnel Questionnaire [7] (*Do you have weakness in your hand or wrist?*), however the focus of this review was to compare performance based outcome measures of muscle weakness.

Assessment of grip strength with dynamometry was the most common method of reporting motor outcome. The usefulness of power grip as an indicator of change in both early (up to 6 weeks post-operatively) and later recovery (>6 weeks) from carpal tunnel release is questionable. Although Simpson [32] states that grip strength is particularly useful to evaluate outcome following carpal tunnel release, she also cautions that it should not be used where tissue healing is incomplete and testing would cause pain. Early post-operative power grip strength showed that values initially decreased and coincides with the assessment of pillar or scar pain or tenderness where higher pain scores were reported in the early post operative phase.

Although none of the studies were designed to investigate an association between pain and grip strength, it is likely that the reduction in grip strength reflects pain inhibition with muscle contraction or increased sensitivity to pressure over the pillar region or scar. Contraction of muscles originating from the flexor retinaculum might cause pain by transmitting tension to the cut and healing transverse carpal ligament or retinaculum. The dynamometer handle may produce discomfort over the scar or pillar region during power grip dynamometry. Ludlow et al [33] comment that whilst grip and post-operative scar tenderness have been shown to predict return to manual work, it is unclear whether these are distinct or whether the discomfort of the handle against the pillar area contributes to low grip results. If the aim is to quantify pain or tenderness to pressure, alternative methods of assessment, such as pressure algometry may be more appropriate [34].

Power grip requires synergistic function of intrinsic and extrinsic muscles of the hand, most of which are supplied by the median nerve proximal to the carpal tunnel, the ulnar or radial nerve [35]. Power grip does not primarily or exclusively use the muscles affected by CTS. Weakness of APB or opponens pollicus (OP) may be masked by compensatory action of synergistic muscles, such as the flexor digitorum superficialis (FDS) and flexor digitorum profundus (FDP), particularly to the ring and little fingers, therefore not significantly reducing power grip. Furthermore, if motor impairment is not marked prior to surgery, the scope for change may also be limited. This is supported by the finding that four [11,25,26,29] of five British studies had pre-operative values within the British normative range for men and women [36] and three [19,20,24] of five North American studies were within the American normative range [30]. The values reported in a Swiss study [13] and a Japanese study [17] fell within the British normative range. Norms are gender and age-specific and therefore comparison of an overall study group mean to norms may be questionable. However, prevalence of CTS increases with age and is higher in women who also have lower normal values than the overall normative group mean. It is unlikely that power grip dynamometry would demonstrate improvement in those with minimal or subtle weakness pre-operatively.

Key pinch or lateral pinch was the most common type of pinch grip assessed. As with power grip, key pinch is a complex motor task involving synergistic muscles. Key pinch strength will be influenced by thumb interphalangeal joint position [32], strength of the 1^st ^dorsal interosseous muscle, innervated by the ulnar nerve, flexor pollicus longus (FPL), innervated by the median nerve proximal to the carpal tunnel and the flexor pollicis brevis (FPB) which has innervation with variable contribution from the median and ulnar nerve. Therefore, as with power grip, APB or OP weakness or pain inhibition may be compensated for during key pinch by synergistic muscle action or 'trick' movements [3]. Tip to tip (thumb pulp to index pulp) and tripod (thumb pulp to index and middle finger pulps) pinch rely more on the thenar muscles and therefore can be argued to be a more appropriate measure of motor involvement in CTS. The responsiveness indices (effect size) for tip pinch show moderate effect sizes (0.33 and 0.58) at 12 weeks after surgery. However, tip pinch values for the pre-operative comparator and experimental groups in Dias et al's study (4.9 kg and 5.7 kg, respectively) were also close to the overall normative range reported by Gilbertson and Barber-Lomax[36]. Women made up 73% of the study sample and the mean age was 56 years. Given that British normative values for women are 4.52 kg (right hand) and 4.53 kg (left hand) and in men 6.79 kg and 6.85 kg, it is likely that for most patients pre-operative tip pinch weakness was minimal. Despite tip pinch targeting more specifically the thenar muscles the scope for improvement is limited and may explain the apparent lack of significant change after surgery. Whilst dynamometry is standardised and has been shown to have high levels of inter- and intra-tester reliability this does not compensate for its apparent lack of validity and ultimately its ability to reflect clinical change in CTS surgery.

Manual muscle testing according to the MRC (Medical Research Council) scale is commonly used by physicians and therapists to evaluate individual muscle strength [37,38]. Theoretically, as MMT can more closely target intrinsic muscles affected by compression of the median nerve in the carpal tunnel, it is more precise in detecting change in motor function due to CTS. Brandsma and Schreuders [37] proposed tests of abduction and opposition to evaluate and monitor the motor function of the muscles innervated by the median nerve. Kuhlman and Hennnessey [39] report APB weakness to have fair to good sensitivity and specificity and recommend that this muscle should be tested in patients with suspected CTS. However MMT has also been criticised for its lack of differentiation especially in the upper ranges of the scale [40,41]. It has been shown that relatively low levels of innervation can produce a grade 4 contraction and that its sensitivity is better in the range of 0 to 3 grades and for diagnosis of nerve deficits [41,42]. The data from Brown et al's [10] study with high pre-operative mean grades of 4.4 and 4.5 indicate that motor weakness was minimal in these patients and would explain why MMT lacks responsiveness to clinical change in this patient group. It is also arguable whether an ordinal scale such as the MRC scale should be summarised using the mean, with the median being more appropriate.

An alternative method for quantifying individual muscle strength including the thenar muscles is the Rotterdam Intrinsic Hand Myometer (RIHM) [43] a hand-held dynamometer which measures in Newtons of force. This device enables quantification of force especially in the higher grades (with 'some' or 'full resistance') thus overcoming the lack of sensitivity of the MRC scale. When testing the intrinsic muscles of the hand it has been shown to have high test-retest reliability (ICC 0.94 or higher) in peripheral nerve injured patients and criterion-related validity has been reported [41]. There are no studies reporting outcome in CTS using this device. Although in this review only few studies utilised MMT with variations in the muscle tested, scale used and classification system, it is possible that MMT is a more specific measure of thenar muscle strength in CTS patients. Further research to evaluate the validity, reliability and responsiveness of measuring the thenar muscles with the RIHM is needed, to establish whether it is a useful outcome measure for the purpose of detecting strength change following surgery for CTS.

Presence or absence of thenar atrophy has not been standardised. It is either recorded as a dichotomous outcome or using a grading scale (e.g. mild, moderate and severe) and the criteria for either have not been defined. It also overlaps to some extent with the assessment of thenar muscles by MMT given that the lowest grade equates to no palpable contraction. It does not meet the criteria of a standardised test and therefore its use as an effectiveness endpoint is questionable.

This review has some limitations. The analysis of results and extraction of information such as patient and device positioning depends on the quality of reporting of the studies. Several studies lacked detail on the methods of assessment and actual scores and had to be excluded from the overall analysis of results. The remaining studies which were included may give a biased result and therefore findings of this review need to be interpreted with caution. The surgical procedure was often described in greater detail than the methods used to assess outcomes. However without this detail it is also difficult to ascertain whether standardised procedures where followed and therefore the extent to which the reader can have confidence in the results. A further limitation was the detail on pre-operative and post-operative scores, which were either not reported at all, given as percentage differences or displayed graphically only. The deficiencies in the reporting of trials has been highlighted in several reviews and has led to the development of Consolidated Standards for Reporting Trials (CONSORT) [44,45]. Widespread adherence to these standards may improve the quality of reporting of future trials including the presentation of statistical results and would facilitate future meta-analysis.

## Conclusion

The assessment of recovery of individual muscle strength and composite functional grip strength has featured in many clinical trials of surgical interventions for CTS. In some cases it has been chosen as the primary outcome measure. If motor function is deemed an important effectiveness endpoint in interventions for CTS then the methods of measuring strength need to be valid, reliable and sensitive to change. Dynamometry for composite power grip and pinch grip has been shown to have high repeatability in both symptomatic and asymptomatic individuals however the mean pre-operative scores often were within or close to normative values suggesting minimal weakness. Pinch grip which is assessed either as lateral (or key) pinch, tip pinch or tripod pinch more specifically targets the thenar musculature and therefore is more specific to median nerve pathology. Effect sizes calculated from published data indicate that tip is more responsive than lateral pinch or power grip. In the latter two types of grip weakness of the thenar muscles can be masked by compensatory action of synergistic muscles unaffected by CTS.

Individual testing of APB and OP by manual muscle strength testing may theoretically be more valid as it targets specific median innervated muscles. However this method is prone to bias due to the lack of sensitivity in the upper grades and subjectivity with which the examiner applies the resistance. The development of a handheld dynamometer which allows quantification of force of individual thenar muscles – the Rotterdam Intrinsic Handheld Myometer (RIHM), may offer an improvement but further research is needed to assess its usefulness as an outcome measure for evaluating changes in muscle strength in patients with CTS.

The use of multiple outcome measures in those trials reviewed reflects the lack of consensus on which method of assessing motor function is the most useful. Using several instruments to assess the same domain increases the burden on patients and the clinicians. A definitive answer to the question of which assessment of motor function is the most valid, reliable and responsive in CTS continues to elude us. However the results of this review would indicate that tip pinch strength assessed by dynamometry using a standardised protocol is currently the most responsive performance based measure for strength and should be used in future clinical trials.

Further research is needed to explore the clinical utility of combining handheld dynamometry with manual muscle testing, for example the RIHM, which would target individual median innervated muscles. Longer-term follow-up data is also needed to evaluate the responsiveness of these tools at more than 12 weeks post intervention.

## Competing interests

The author(s) declare that they have no competing interests.

## Authors' contributions

JG contributed to data extraction, interpretation of the pinch measurement data and drafted the manuscript. RC contributed to data extraction, interpretation of the grip strength data, and statistical analysis. SK contributed to the data extraction, interpretation of the manual muscle testing, thenar atrophy data and statistical analysis. CJH conceived the original idea, concept and design of the review and critically revised the manuscript. All authors read and approved the final manuscript.

## Pre-publication history

The pre-publication history for this paper can be accessed here:


